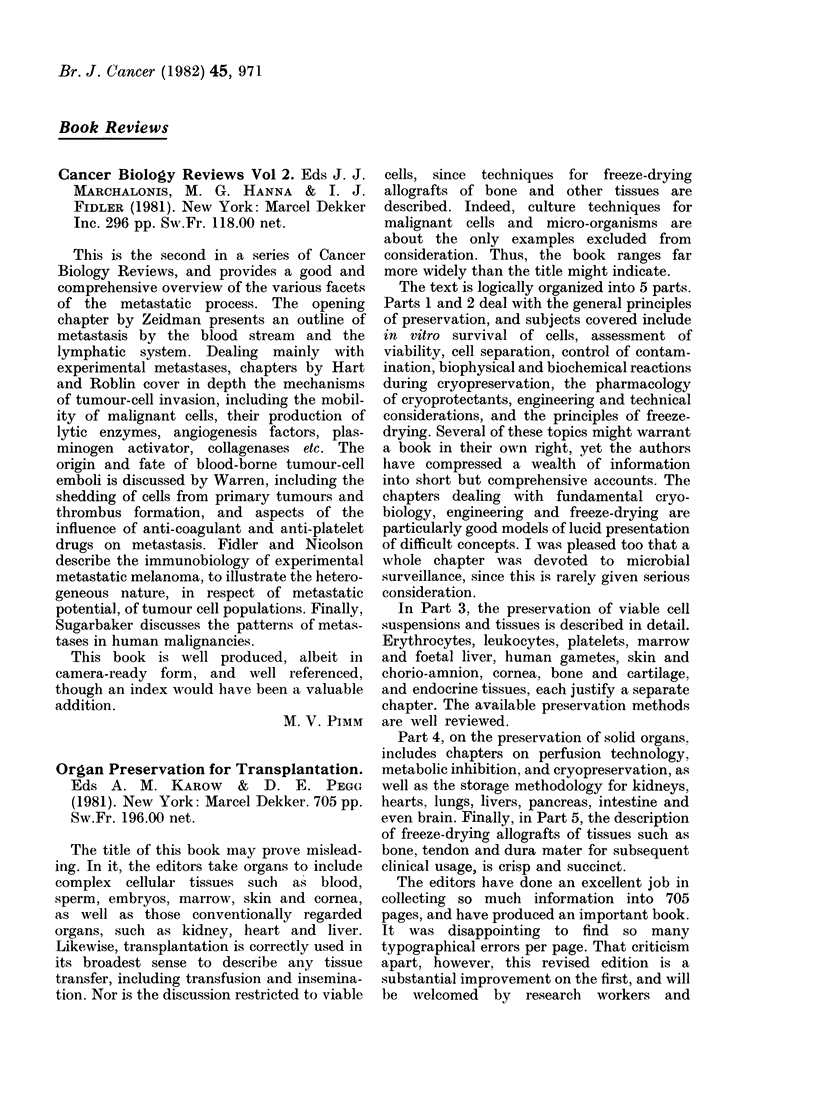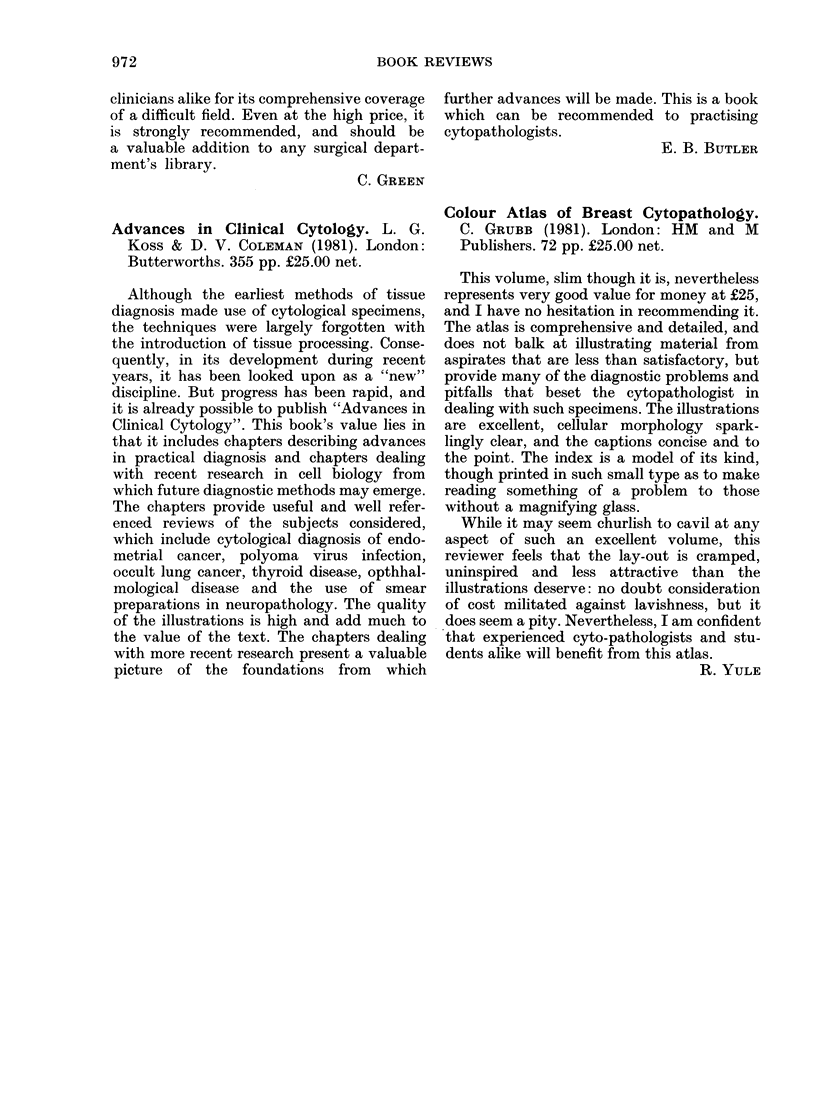# Organ Preservation for Transplantation

**Published:** 1982-06

**Authors:** C. Green


**Organ Preservation for Transplantation.** Eds A. M. Karow & D. E. Pegg (1981). New York: Marcel Dekker. 705 pp. Sw.Fr. 196.00 net.

The title of this book may prove misleading. In it, the editors take organs to include complex cellular tissues such as blood, sperm, embryos, marrow, skin and cornea, as well as those conventionally regarded organs, such as kidney, heart and liver. Likewise, transplantation is correctly used in its broadest sense to describe any tissue transfer, including transfusion and insemination. Nor is the discussion restricted to viable cells, since techniques for freeze-drying allografts of bone and other tissues are described. Indeed, culture techniques for malignant cells and micro-organisms are about the only examples excluded from consideration. Thus, the book ranges far more widely than the title might indicate.

The text is logically organized into 5 parts. Parts 1 and 2 deal with the general principles of preservation, and subjects covered include *in vitro* survival of cells, assessment of viability, cell separation, control of contamination, biophysical and biochemical reactions during cryopreservation, the pharmacology of cryoprotectants, engineering and technical considerations, and the principles of freeze-drying. Several of these topics might warrant a book in their own right, yet the authors have compressed a wealth of information into short but comprehensive accounts. The chapters dealing with fundamental cryobiology, engineering and freeze-drying are particularly good models of lucid presentation of difficult concepts. I was pleased too that a whole chapter was devoted to microbial surveillance, since this is rarely given serious consideration.

In Part 3, the preservation of viable cell suspensions and tissues is described in detail. Erythrocytes, leukocytes, platelets, marrow and foetal liver, human gametes, skin and chorio-amnion, cornea, bone and cartilage, and endocrine tissues, each justify a separate chapter. The available preservation methods are well reviewed.

Part 4, on the preservation of solid organs, includes chapters on perfusion technology, metabolic inhibition, and cryopreservation, as well as the storage methodology for kidneys, hearts, lungs, livers, pancreas, intestine and even brain. Finally, in Part 5, the description of freeze-drying allografts of tissues such as bone, tendon and dura mater for subsequent clinical usage, is crisp and succinct.

The editors have done an excellent job in collecting so much information into 705 pages, and have produced an important book. It was disappointing to find so many typographical errors per page. That criticism apart, however, this revised edition is a substantial improvement on the first, and will be welcomed by research workers and clinicians alike for its comprehensive coverage of a difficult field. Even at the high price, it is strongly recommended, and should be a valuable addition to any surgical department's library.

C. Green